# Data of numerical simulation and experimental research on the design of a cyclone separator with a high flux density

**DOI:** 10.1016/j.dib.2018.08.210

**Published:** 2018-09-07

**Authors:** Nikolay Mikheev, Ilya Saushin, Anna Goltsman, Victor Fafurin

**Affiliations:** a Federal State Budgetary Institution of Science “Kazan Scientific Center of the Russian Academy of Sciences”, 2/31, Lobachevsky St., PO Box 261, Kazan, Tatarstan 420111, Russia; b Federal State Unitary Enterprise “All-Russian Research Institute of Flow Metering”, 7 A, Vtoraya Azinskaya St., Kazan, Tatarstan 420088, Russia

## Abstract

This article contains data related to the research article entitled “Cyclone separator for gas-liquid mixture with high flux density” (Mikheev et al., 2018 [1]doi: 10.1016/j.powtec.2018.08.040). This work was aimed at the development of an effective gas-liquid cyclone separator at a high flux density. Within the implementation of the project, the goal was to choose the schematic construction of the separator provides the reduced dynamic head of flow in the zones with increased liquid phase concentration to prevent the entrainment of separated liquid and layer losses. The paper presents the numerical simulation data and experimental data allowed estimation of separation efficiency of the separator.

**Specifications table**

**Table:** 

Subject area	*Fluid Mechanics*
More specific subject area	*Multiphase Flow*
Type of data	*Table, text file, graph, figure*
How data was acquired	*FLUENT 18.2 for numerical analysis, ultrasonic flow meter IRVIS RS4-Ultra,liquid weigh-scale CAS AD-2.5, high-accuracy differential pressure transmitter Keller PD-41 ×*
Data format	*Raw data and analysed*
Experimental factors	*The geometric model and the boundary conditions for setting the problem of numerical simulation are completely based on the full-size model of the separator. Experiments were performed on a scaled 1:2 model of the cyclone separator. The reproduction of the flow regimes in the scale model was carried out by increase in the volumetric flow rate through the separator when several dimensionless numbers of separation processes in the cyclone are maintained.*
Experimental features	*For the numerical model, three-dimensional Reynolds-averaged Navier-Stokes (RANS) equations and the continuity equation were solved using FLUENT 18.2 which employed the control-volume technique and the Semi-Implicit Method for Pressure-Linked Equations (SIMPLE) velocity-pressure coupling algorithm with the second order upwind discretization. Reynolds stress turbulence model.*
	*Cyclone separation efficiency was estimated using a specially designed test bench. Prior to the experiment, dry air was blown through the system. The air flow rate and pressure were adjusted by the valve opening and measured by an ultrasonic flow meter IRVIS RS-4 Ultra. After the test bench had reached steady operation regime, constant liquid mass flow rate was supplied to the system in front of the separator for a fixed time interval. The volume of liquid collected by the separator was evaluated by weighing results. Separation efficiency was estimated with correction for water loss due to saturation of dry air with water vapor. The correction was calculated using the maximum amount of water in air according to DIN ISO 7183. Flow resistance coefficient was estimated from the ratio of the measured static pressure drop across the cyclone (in its inlet and outlet ducts) to the dynamic head calculated using the mean velocity in the ducts.*
Data source location	*Kazan, Russia*
Data accessibility	*Data is with this article*
Related research article	*Mikheev N, Saushin I, Paereliy A, Kratirov D, Levin K. Cyclone separator for gas-liquid mixture with high flux density. Powder Technology. 2018 339:326-333.*doi: 10.1016/j.powtec.2018.08.040

**Value of the data**

• Numerical simulation and experimental data can be used by designers and engineers in the development of gas-liquid cyclone separators with high flux density. The presented data are sufficient for the reproduction of numerical simulation in other laboratories.
• The numerical and experimental data can be used to test different turbulence models, boundary conditions, mesh design, discretization scheme, steady-state and transient simulations etc.
• The design, method and results of an experimental study of a separator with a pressure in its body much higher than atmospheric represent scientific and practical relevance and can be easily reproduced in other laboratories.

## 1 Data

The data presented in this article is based on simulation of the gas-liquid mixture flow with high flux density in the cyclone body [1], [2] (Supplementary 1 Geom.stp), which was conducted using Computational Fluid dynamics (CFD) modelling and experimental testing. Geometric model of the separator is designed in KOMPAS 15.0 software and slightly simplified in ANSYS DesignModeler software for numerical simulation. In particular, hemispherical shapes of the upper and lower bottoms of the cyclone body were simplified.

Detailed fields of characteristics that could be the cause of entrainment [3] and "layer losses" [4] significantly reduced separation efficiency are based on numerical simulation of single-phase gas medium flow in cyclone body. These data allow us to estimate the values of the dimensionless numbers used to analyze the separation efficiency in [5], [6] Standards. In particular, the value of the dynamic head of the gas flowing around the walls of the cyclone body in the zones with liquid phase concentration and the *K*-value parameter from Souders-Brown equation [7]

(1) $$K=V_{g}{\left(\frac{\rho_{g}}{\rho_{l}-\rho_{g}}\right)}^{2/3},$$

where *ρ*_l_ и *ρ_g_* are respectively the liquid and gas density, *V_g_* is the velocity of gas.

The experimental data of the gas-liquid mixture flow through a scale model of a separator show the influence of mass flow on the Stokes, Reynolds, and Euler numbers, and theirs conservation law is often used to scale the design of the separator when the required operating pressure in the body changes. The estimation of water loss due to saturation of dry air with water vapor as a function of the specific density is presented.

## 2 Experimental design, materials, and methods

### 2.1 Numerical design and data collection method

The commercial ANSYS Fluent 18.2 numerical code was used for predicting the flow in the cyclone body. The numerical code was used to solve the Reynolds averaged Navier–Stokes equation (RANS simulation) which employed a control-volume-based technique. The Reynolds stress turbulence model was used within the model second-order upwind scheme was used to discretize all the transport equations. The numerical code used the semi-implicit method for pressure-linked equations (SIMPLE) algorithm for the velocity–pressure coupling of the computation. The selected resolution of the grid was based on the grid-sensitivity analysis.

**Fig. 1:**
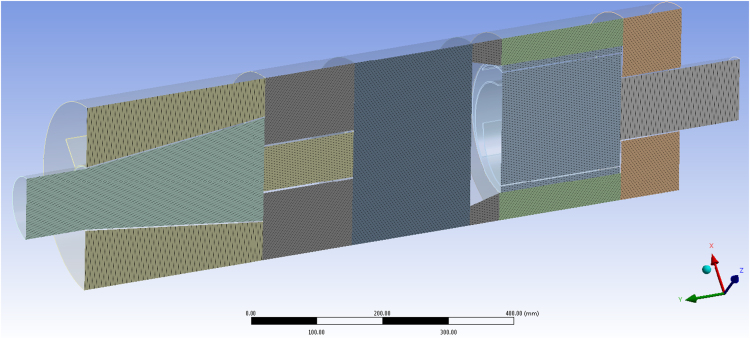
Geometric model of separator divided into trivial geometric bodies.

**Fig. 2:**
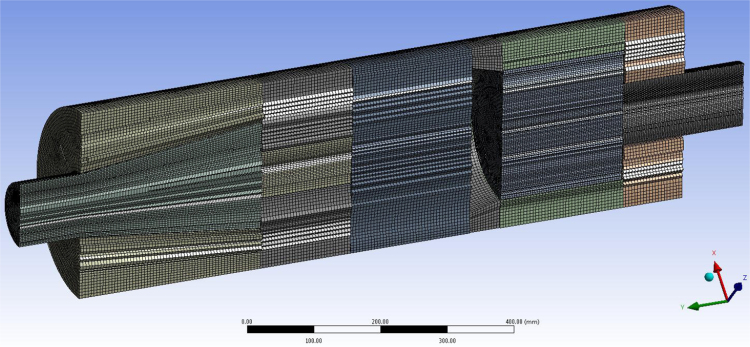
Computational mesh.

To construct a structured mesh, the geometric model was divided into several trivial zones, which were extrusion bodies (Fig. 1). It made possible to simplify considerably the construction process and reduce the number of elements of the computational grid. The complete meshed model comprised of 1.3 million elements (Fig. 2). The computations were performed using parallel processing on a workstation with one Amd Fx 8320 eight-core 3.5 GHz processor and 16 GB Fully Buffered DDR3. The simulations were completed after additional iterations showed no further variation in the velocity results.

**Fig. 3:**
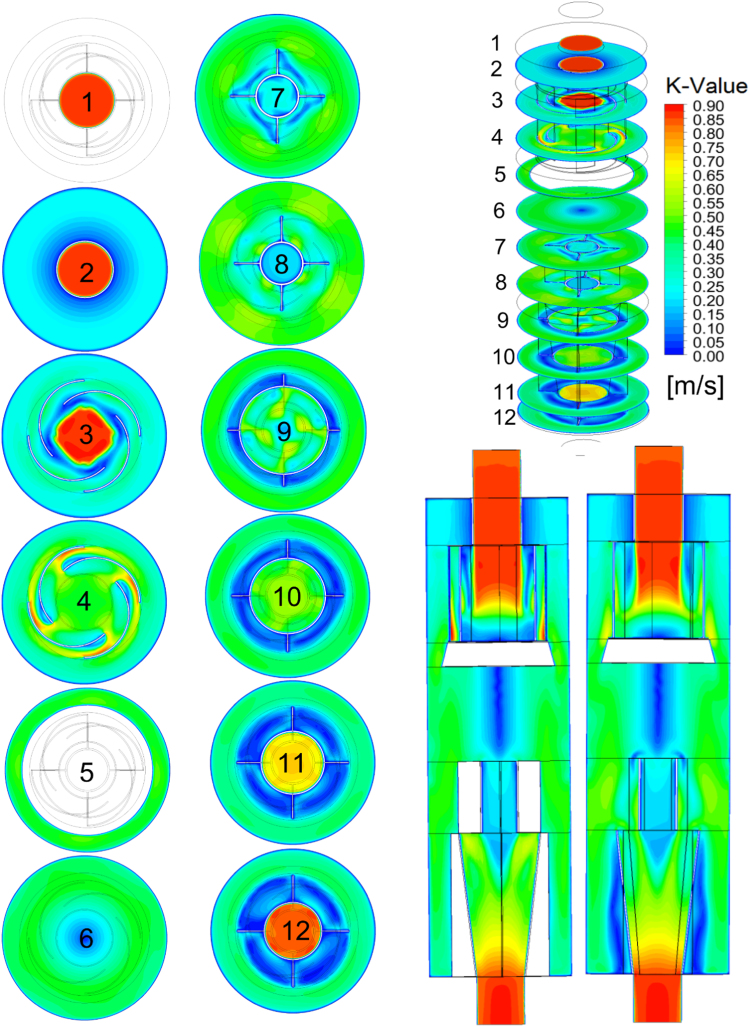
*K*-value field.

**Fig. 4:**
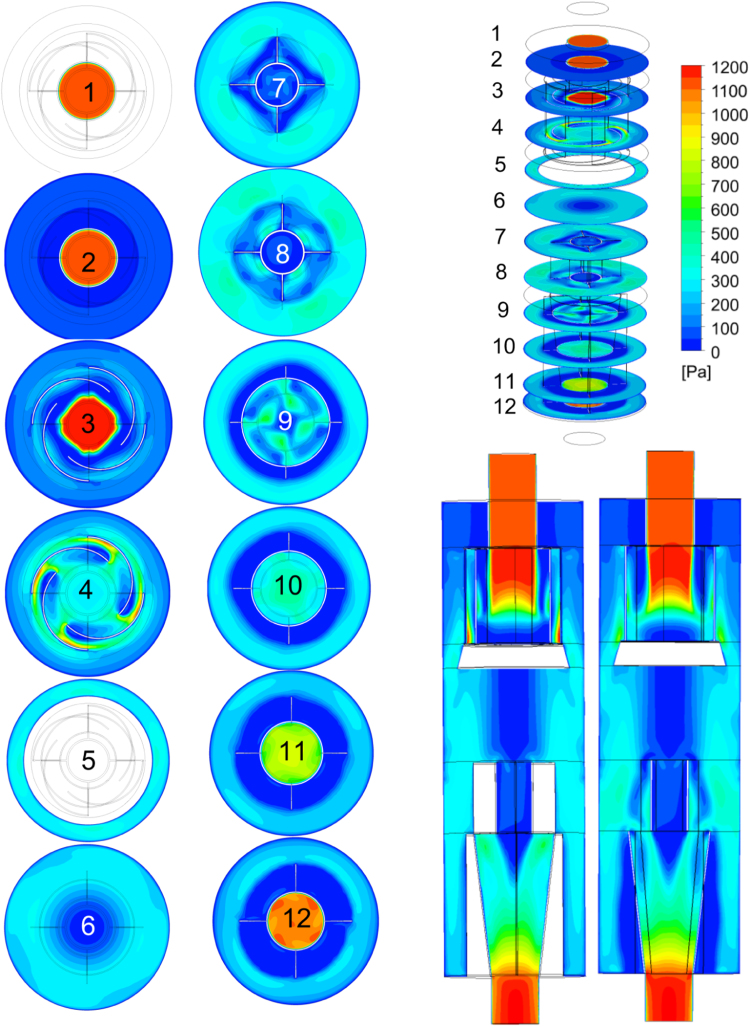
Dynamic head of gas field.

Fig. 3, Fig. 4 show, respectively, the detailed fields of the K-Value and dynamic head of gas in the cyclone body.

### 2.2 Experimental design and data collection method

**Fig. 5:**
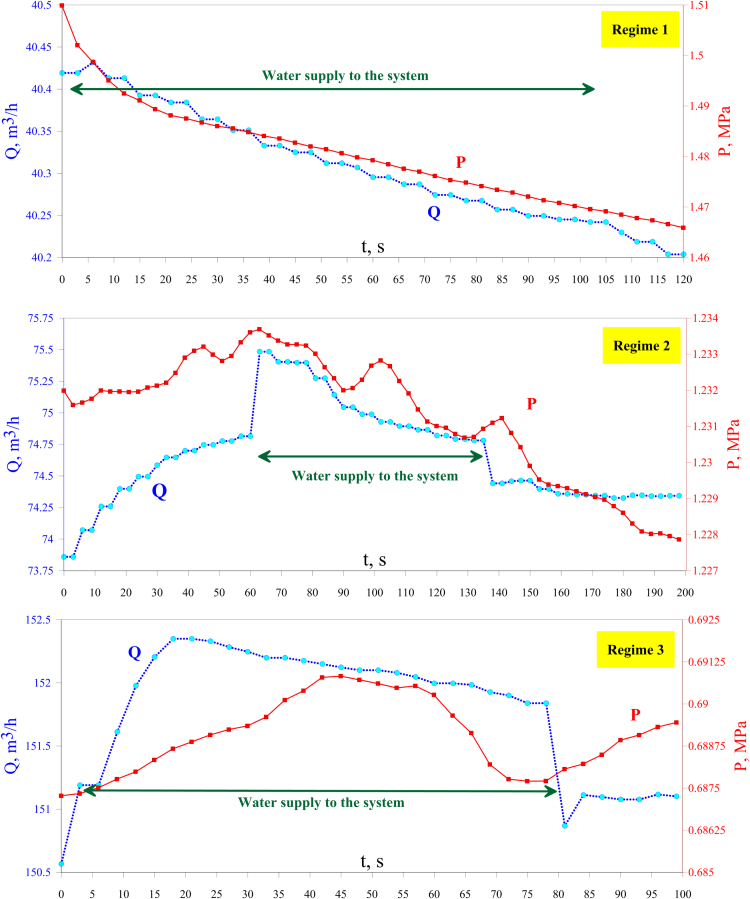
Flow rate behavior and dry air pressure variation.

Cyclone separation efficiency was estimated using a specially designed test bench. The experiments were performed on a scaled 1:2 model of the considered cyclone for three different separation regimes (at different pressures). The maximum allowable operating pressure in the test bench was 1.6 MPa, therefore the required flus density was attained by increase in the volumetric flow rate. Air was employed as a gas phase while water was used as a liquid phase. Prior to the experiment, dry air was blown through the system. Then the tank was filled with 2 kg of water through the filler neck valve at the preparation step. The weight of the water was measured by means of a CAS AD-2.5 weigh-scale with a calibration interval of 0.5 g. The air flow rate and pressure were adjusted by the valve opening and measured by an ultrasonic flow meter IRVIS RS-4 Ultra. The water loss due to dry air saturation with water vapor was calculated using the maximum amount of water in air (according to [8]). Static pressure drop, ∆P, across the cyclone was measured using a high-accuracy differential pressure transmitter Keller PD-41 X with inaccuracy of 0.1%. Fig. 5 show the pressure and volume flow rates of dry air in the system measured by the flowmeter. The (Supplementary Table 1) contains the full logs of the measurement data of the flowmeter, where

– time, s – time;
– *T*, K – temperature;
– *P*, kPa – static pressure;
– *U*, m/s – mean velocity;
– *Q_oc_*, m^3^/h – volumetric gas flow rate at operating conditions;
– *Q_nc_*, m^3^/h – volumetric gas flow rate at normal conditions.
